# Addressing the issue of surface mechanisms and competitive effects in Cr(VI) reductive-adsorption on tin-hydroxyapatite in the presence of co-ions

**DOI:** 10.1038/s41598-023-44852-7

**Published:** 2023-11-02

**Authors:** Tiziana Avola, Sebastiano Campisi, Laura Polito, Silvia Arici, Ludovica Ferruti, Antonella Gervasini

**Affiliations:** 1 Dipartimento di Chimica, Università degli Studi di Milano, Via Camillo Golgi 19, 20133 Milan, Italy; 2 Istituto di Scienze e Tecnologie Chimiche “Giulio Natta”, SCITEC-CNR, Via G. Fantoli 16/15, 20138 Milan, Italy; 3 A2A Ciclo Idrico S.P.A., Laboratorio Chimico, Via Lamarmora, 230, 25124 Brescia, Italy; 4 A2A S.P.A, Group Risk Management, Enterprise Risk Management, C.so di Porta Vittoria, 4, 20122 Milan, Italy

**Keywords:** Environmental sciences, Chemistry, Materials science

## Abstract

Our group recently proposed an innovative sustainable reductant-adsorbent material, tin(II)-hydroxyapatite (Sn/HAP, *ca*. 10 wt% Sn) for the interfacial Cr(VI) reductive adsorption process. In this study, Cr(VI) removal capacity was evaluated in multi-component solutions containing representative background ions (i.e., CaCl_2_, Ca(NO_3_)_2_, MgSO_4_, Na_2_SO_4_, Fe(NO_3_)_3_, AlCl_3_, Zn(NO_3_)_2_, or Mn(NO_3_)_2_). Sn/HAP was able to reduce Cr(VI) with complete Cr^3+^ adsorption on HAP surface, except in the presence of Fe^3+^ and Al^3+^ ions. Some metal ions co-existing in solution, such as Fe^3+^, Al^3+^, Zn^2+^, and Mn^2+^, were also adsorbed on HAP surface. Reuse experiments of the Sn/HAP sample, up to 7 runs, resulted in a total amount of reduced Cr(VI) of *ca.* 15–18 mg g^−1^. Fast kinetics of Cr(VI) reductive adsorption at 25 °C in a multi-metal component solution was observed. The *pseudo-second order* model was in excellent agreement with the experimental kinetic data, leading to a rate constant (*k*_25°C_) value of *ca*. 30 M^−1^ s^−1^. The collection of adsorption isotherms of Cr^3+^ and Fe^3+^, together with TEM–EDX analysis permitted the unveiling of competitive adsorption phenomena between metal ions. The obtained results demonstrate that Sn/HAP could be an efficient material for the removal of hexavalent chromium in aqueous solutions containing high concentrations of inorganic impurities.

## Introduction

The reductive adsorption process has emerged as a promising approach for Cr(VI) remediation, as it combines Cr(VI) reduction with contextual Cr^3+^ immobilization by adsorption onto a suitable adsorbent^1, 2^. So far, several materials have been identified in the literature as potential solid-phase reductants/adsorbents, e.g., iron minerals (magnetite, goethite, biotite)^3, 4^ or other iron-based materials^5–10^, as well as polymeric composite materials^11–14^, inorganic and metallic nanoparticles^15–19^, hybrid nanomaterials^20–23^, and metal-organic frameworks^24–26^.

Recently, in the context of green chemistry and circular economy concepts, the environmental friendliness of materials used as reducing agents/adsorbents has become an important factor to be considered^27–29^. In this context, Sn-functionalized hydroxyapatite (Sn/HAP) has proved to be an effective and environmentally friendly solid-phase Cr(VI) reductant/adsorbent^30, 31^. The characterization study revealed that the reductive adsorption process took place on the surface of Sn/HAP according to a two-step mechanism, which involves an interfacial combination of Cr(VI) reduction by Sn^2+^ sites followed by Cr^3+^ formation/adsorption on HAP surface. A homogeneous Sn^2+^ dispersion onto hydroxyapatite surface, as observed by High-angle annular dark-field scanning transmission electron microscopy (HAADF-STEM), Energy Dispersive X-ray (EDX) and X-ray Photoelectron Spectroscopy (XPS) analyses, produced an effective Cr(VI) reduction/Cr^3+^ adsorption up to *ca*. 20 mg_Cr_ g^−1^ with Sn loading of 15 wt%, even at non-acidic pH^31^. This adsorption capacity was already compared with that obtained with some other ecofriendly materials in our previous paper^30^. It emerges that it is comparable to or higher than that of activated carbon (13.7 mg_Cr_ g^−1^), biochar (14.8 mg_Cr_ g^−1^), alumina (8.5 mg_Cr_ g^−1^), kaolinite (7.8 mg_Cr_ g^−1^), boehmite (19.8 mg_Cr_ g^−1^) or mordenite (10.9 mg_Cr_ g^−1^). On the other hand, synthetic adsorbents, such as resins or zeolites, can assure remarkable adsorption capacity (up to 200 mg_Cr_ g^−1^): however, their widespread use could be restricted due to their high cost, environmental impact and problems in the disposal of spent adsorbents^32^.

Compared with other materials, zero valent iron-based materials (ZVI) have many advantages such as the strong oxidizing ability to ferrous/ferric iron, the possibility to retain the formed Cr^3+^ through multiple mechanisms (adsorption, co-precipitation, and precipitation)^5^. Furthermore, the immobilization of ZVI nanoparticles onto porous inorganic materials (e.g. silica^33^ or magnesium hydroxide^34^) or natural polymeric materials (lignin, chitosan)^35^ allows to improve their stability and offers additional adsorption capacity towards metal cations, achieving chromium uptake up to 150 mg_Cr_ g^−1^. However, according to Fang et al.^5^, who recently provided an exhaustive overview of zero valent iron-based materials for the sequestration of aqueous Cr(VI), the main barriers in market penetration are the high production costs and the toxicological effects.

From this point of view, Sn/HAP emerges as an interesting non-conventional ‘low-cost’ and environmental-friendly adsorbent. In fact, according to a recent ranking proposed in the literature, tin has a higher environmental friendliness index compared to iron, based on parameters such as toxicity, endangerment degree and life cycle assessment (LCA)^36^. On the other hand, hydroxyapatite is a non-toxic material that can be extracted from waste or synthesized from cheap feedstock (with an estimated production cost of about 1.25 USD/kg)^32^.

In addition, we have recently demonstrated that after use as reductive adsorbents for Cr(VI) removal, Sn/HAP can be successfully upcycled into catalyst in gas-phase catalytic processes for air pollution remediation, thus resulting in a more eco-efficient disposal practice.

For this reason, Sn/HAP materials deserve to be further investigated, by exploring the effects of background circumstances and the influence of co-existing ions on the reductive adsorption of Cr(VI) by Sn/HAP. In fact, anions (such as chloride, nitrate, and sulphate) and cations (such as Ca^2+^, Mg^2+^, Na^+^, Zn^2+^, Mn^2+^, Fe^3+^ and Al^3+^) that commonly coexist in polluted aqueous environment can (i) increase the ionic strength of aqueous solutions, (ii) adsorb on the Sn/HAP surface, and (iii) change the thickness and interfacial potential of the double layer and/or (iv) exhibit competing effects depending on their nature and properties. If these ions coexist in the aqueous solution, they may affect the reductive adsorption behaviour of Sn/HAP and the fate of the target contaminant Cr(VI).

Therefore, as an extension of previous work, the competing effects of co-ions were investigated in this work, with the prospect of testing a real-world application for pollutant remediation. In addition, the sustained activity of Sn/HAP over several reductive adsorption runs was explored.

## Experimental section

### Materials

Aqueous solutions of calcium nitrate tetrahydrate, Ca(NO_3_)_2_·4H_2_O (> 99.0%, Merck ACS) and diammonium hydrogen phosphate, (NH_4_)_2_HPO_4_ (> 98.0%, Sigma-Aldrich) were used as precursors for the synthesis of hydroxyapatite, ammonium hydroxide solution, NH_4_OH (28–30 wt%, Fluka) was used for pH adjustment during the synthesis.

Tin chloride dihydrate (SnCl_2_·2H_2_O, ≥ 98.0% oxidimetric assay), potassium bichromate (K_2_Cr_2_O_7_, ≥ 99.0% oxidimetric assay), calcium nitrate tetrahydrate, (Ca(NO_3_)_2_·4H_2_O, > 99.0%, ACS), magnesium sulfate anhydrous (MgSO_4_, 99%) and zinc nitrate hexahydrate (Zn(NO_3_)_2_·6H_2_O, for analysis) were purchased from Carlo Erba. Calcium chloride (CaCl_2_, ≥ 99.99%), sodium sulfate decahydrate (NaSO_4_·10H_2_O, ≥ 99.0%, ACS Reagent), iron(III) nitrate nonahydrate (Fe(NO_3_)_3_·9H_2_O, ≥ 99.95%) and manganese nitrate tetrahydrate (Mn(NO_3_)_2_·4H_2_O, ≥ 97.0%) salts were purchased from Sigma-Aldrich. Aluminum chloride hexahydrate (AlCl_3_·6H_2_O, 99%) was from Fluka. Hydrochloric acid (37 wt%) was from Merck. Nitrogen, 99.9995% purity from SAPIO was used as inert gas. All the solutions were prepared using MilliQ water (ρ ≥ 17.5 MΩ cm; TOC, 2 ppb).

Tin-functionalized hydroxyapatite (Sn/HAP, 10 wt% Sn loading) was prepared from an acidic tin chloride solution (pH ~ 2) by using a flash deposition technique already validated to deposit Sn^2+^ species onto HAP^31^.

### Characterization

Sn loading of Sn/HAP samples was determined by Inductively Coupled Plasma Mass Spectrometry (ICP-MS) technique by using an iCAP Q ICP-MS (Thermo Fischer Scientific), equipped with an ASX-560 Autosampler. Prior to the analysis, a weighted amount of Sn/HAP powder was digested in 3 mL HCl 37% and 1 mL HNO_3_ at 110 °C for two hours.

Sn/HAP samples were characterized by means of ZEISS LIBRA 200FE microscope with a 200 kV FEG source, in column second-generation omega filter for Transmission Electron Microscopy (TEM). High angular annular dark field scanning transmission electron microscopy (HAADF-STEM) facility and Energy-dispersive X-ray (EDX) probe (Oxford INCA Energy TEM 200) were employed for the chemical analysis of the samples.

### Computation

Visual MINTEQ software (ver 3.1) was used to compute metal speciation, solubility equilibria, sorption, and so on for natural waters (available online: https://vminteq.lwr.kth.se/)^37^. The chemical compositions reported in Table S.2 (see Supporting Information) were input into the software to simulate the metal species distribution as a function of pH at temperature of 25 °C.

### Hexavalent chromium reductive adsorption tests

The tests of reductive adsorption of Cr(VI) were carried out at 25.0 ± 0.5 °C in binary aqueous solutions containing K_2_Cr_2_O_7_ salt, to obtain nominal concentration of Cr(VI) of 20 mg L^−1^, and another metal salt present in defined amount. The nature of salts was chosen starting from the composition of a real groundwater sample kindly furnished from A2A Company, Brescia (BS), Italy (Table S.1).

To realize the tests, a given amount of dried Sn/HAP powder (ca. 0.2 g) was placed in test tubes containing 40 mL of Cr(VI) solution (20 mg L^−1^) in co-presence of a single salt among those selected for the present study. Table S.2 reports the used initial concentrations of each anion/cation. The pH value was adjusted to 2.0 by adding 4 mL of HCl 0.1 M, resulting in a final dosage of 4.5 g L^−1^ (0.2 g Sn/Hap in 44 mL solution) to guarantee the complete solubilisation of all salt components. The suspensions were maintained at 25 °C under magnetic stirring for 2 h. At the end of the tests, sample tubes were centrifuged at 5000 rpm for 5 min, then the supernatant liquid was recovered to determine residual concentrations of both Cr(VI) and the other metal cation. All the reductive adsorption experiments were performed in duplicate.

Reuse tests were carried out after recovering the Sn/HAP sample (after use in the first test) by centrifugation (5000 rpm for 5 min); consecutive tests (up to seven times) under the same experimental conditions of the first one were carried out in the same test tubes containing the Sn/HAP sample. During the last reduction test, the suspension was maintained at 25 °C under magnetic stirring for 4 h, to make sure the equilibrium was reached. The initial and final pH values have been measured and gathered in Table S.3

Kinetic tests of Cr(VI) reductive adsorption were carried out at 25.0 ± 0.5 °C in independent batch reactors containing 20 mg L^−1^ of Cr(VI) and all the other studied metal cations at generated pH solution of 2.8 ± 0.2. The initial concentrations used are listed in Table S.2. In this case, nitrate salts were used as metal precursors to avoid precipitation of poor soluble metal sulphates and/or chlorides. The reaction was stopped after 3, 10, 30, 50, 100 min by filtration and separation of Sn/HAP powder from each reactor, and the residual Cr(VI) concentration in supernatant solutions was determined by spectrophotometric analysis (see method reported in the paragraph *Analytical Methods*).

Percent removal efficiency, *η* (%), and removal capacity, *q* (mg g^−1^) of Cr(VI), Cr^3+^, and the other metal ion (Me) present in solution were calculated to evaluate the performance of Sn/HAP, by using the following Eq. (1) and Eq. (2), respectively:

1 $${\text{Removal efficiency}},\eta \left( {\text{\%}} \right) = { }\left( {\frac{{C_{0} - C_{f} }}{{C_{0} }}} \right){*}100$$

2 $${\text{Removal capacity}},q \left( {{\text{mg}} \cdot {\text{g}}^{ - 1} } \right) = \frac{{\left( {C_{0} - C_{f} } \right){*}V_{sol} }}{{m_{ads} }}$$

where *C*_*0*_ represents the initial Cr(VI) or Me concentrations (mg L^−1^), determined by spectrophotometric detection or by ICP-MS, respectively; *C*_*f*_ represents the final Cr(VI) or Me concentrations (mg L^−1^), determined by spectrophotometric detection or by ICP-MS, respectively; *V*_*sol*_ (L) is the volume of solution; *m*_*ads*_ is the mass of Sn/HAP (g). In the case of Cr^3+^, *C*_*f*_ is the final concentration of Cr^3+^ calculated by difference between Cr(VI) (determined by spectrophotometric analysis) and total chromium residual concentration, Cr(VI) + Cr^3+^ (determined by ICP-MS).

In the kinetic study, the Cr(VI) removal capacity at different times *t* (*q*_*t*_, mg g^−1^) was calculated by the Eq. (3):

3 $${q}_{t}=\frac{\left({C}_{0}-{C}_{t}\right)*{V}_{sol}}{{m}_{ads}}$$

where *C*_*0*_ and *C*_*t*_ are the Cr(VI) concentrations (mg L^−1^) at *t* = 0 and *t* = *t*, respectively; *V*_*sol*_ (L) and *m*_*ads*_ (g) are the same as described above.

Three commonly used kinetic models were chosen to fit the experimental data of the kinetics of Cr(VI) reductive adsorption reaction, i.e. the pseudo-first order (PFO), pseudo-second order (PSO) and Elovich models. As shown in the following Eqs. (4)–(6), kinetic equations have been used in their respective integrated linearized forms^38^:

4 $${\text{PFO}}\,\ln \left( {q_{e} - q_{t} } \right) = \ln q_{e} - k_{1} t$$

5 $${\text{PSO}}\,\frac{t}{{q_{t} }} = \frac{1}{{k_{2} q_{e}^{2} }} + \frac{t}{{q_{e} }}$$

6 $${\text{Elovich}}\,q_{t} = \frac{1}{b}\ln \left( {ab} \right) + \frac{1}{b}\ln t$$

where *q*_*e*_ and *q*_*t*_ are the adsorbed amount of Cr(VI) per unit mass of Sn/HAP (mg g^−1^) at adsorption equilibrium and at time *t*, respectively; *k*_*1*_ (min^−1^) and *k*_*2*_ (g mg^−1^ min^−1^) are the pseudo-first order and pseudo-second order rate constants, respectively; *a* and *b* are the Elovich constants, i.e. the first represents the initial rate constant and the latter accounts for the surface coverage and/or activation energy of the adsorption.

### Adsorption isotherms of Fe^3+^ and Cr^3+^

Isotherms of adsorption of the trivalent cations, Fe^3+^ and Cr^3+^, onto Sn/HAP (0.1 g) were carried out at constant temperature (25.0 ± 0.5 °C), at constant pH (ca. 2.0 ± 0.2), and at constant dosage (4.5 g L^−1^). Each suspension was maintained under magnetic stirring for a total time of 2 h to achieve equilibrium^39^.

To collect each isotherm, different test tubes with increasing initial metal ion concentration of 200–3000 mg L^−1^ and 50–3000 mg L^−1^ for Fe^3+^ and Cr^3+^, respectively, were prepared. After two hours, the supernatant solution was recovered by centrifugation (5000 rpm for 5 min) and the residual Fe^3+^ or Cr^3+^ concentration was measured by ICP-MS (see paragraph *Analytical Methods*).

The Langmuir isotherm model was used to process the experimental data of adsorption equilibria (*q*_*e*_ vs. *C*_e_), by using the corresponding non-linear equation, Eq. 7^40^:

7 $${q}_{e}=\frac{{q}_{max}{K}_{L}{C}_{e}}{1+{K}_{L}{C}_{e}}$$

where *q*_*max*_ (mg g^−1^) is the maximum adsorption capacity of Sn/HAP, *C*_*e*_ (mg L^−1^) is the metal ion concentration at equilibrium and *K*_*L*_ (L mg^−1^) is the Langmuir constant, reflecting the affinity of the adsorbent material towards the interested species.

### Analytical methods

Metal cation concentration and total concentration of Cr were determined by Inductively Coupled Plasma Mass Spectrometry (ICP-MS, *UNI EN ISO 17294–2 method*) by using an iCAP Q ICP-MS (Thermo Fischer Scientific), equipped with an ASX-560 Autosampler. Ion Chromatography technique (*EPA 300.0 part A method*) was used to determine anions concentration. IC apparatus (Eco IC model, Metrohm) was constituted by a Metrosep A Supp 10-250/4.0 column and equipped with a Compact Autosampler Metrohm. Software MagIC Net Basic (Version 4.0 Build 137) was used for data acquisition.

Initial and final Cr(VI) concentrations were determined by both spectrophotometric analysis (*APAT CNR IRSA 3150C method*) and Ion Chromatography (*EPA218.7 method*), according to the concentration magnitude. UV–vis spectrophotometric analysis was performed by using a Shimadzu spectrophotometer (UV 1900 model), working at 540 nm by using 1,5-diphenyl carbazide (DPC) method. IC apparatus was constituted by a Metrosep A Supp 10 250/4.0 column, a 6.2836.000 Post-column reactor (Metrohm) and a 944 Professional UV/VIS Detector Vario (Metrohm), working at 538 nm. Software MagIC Net Compact (Version 4.0 Build 137) was used for data acquisition.

Amounts of Cr, Fe, Al, Zn, Mn cations, immobilized on the used Sn/HAP samples, were also determined by ICP-MS after solid digestion performed in 3 mL HCl 37% and 1 mL HNO_3_ at 110 °C for two hours.

## Results and discussion

Recent studies have shown that Sn-functionalized hydroxyapatite (Sn/HAP) is an efficient material for reductive adsorption of Cr(VI)^30, 31^. An optimal Sn loading around 10 wt% assured the complete removal of 20 mg g_Sn/HAP_^−1^ of Cr(VI). In addition, HAADF-STEM/EDX mapping and XPS analysis revealed that a high Sn-dispersion at the HAP surface was responsible for an effective interfacial reduction of Cr(VI) with simultaneous adsorption of formed Cr^3+^ and XRD showed the crystalline pattern typical of the sole presence of HAP^30^. The reductive adsorption of Cr(VI) occurred at solid/liquid interphase as confirmed by leaching tests; less than 1 mol% of the total amount of Sn^2+^ present on the solid was released in solution, proving the stable anchoring of these species at HAP surface under working conditions. From a practical point of view, Sn/HAP demonstrated remarkable activity in a wide range of pH (from 2 to 9) and even in oxidant atmosphere.

Here, the reductive activity of Sn/HAP towards Cr(VI) was evaluated in co-presence of several anions and cations typically present in polluted waters to investigate possible interference effects and competition for adsorption by co-ions in the removal of Cr(VI).

Different batches of Sn/HAP were prepared with a nominal tin loading of 10 wt% by a flash deposition technique, consisting in a short contact time during HAP functionalization with SnCl_2_^41, 42^. The actual tin loading (average value, 13.39 wt%) and (Ca + Sn)/P molar ratio (average value, 2.27) were determined by ICP-MS and listed in Table S.4. The value of the (Ca + Sn)/P molar ratio is higher than the stoichiometric Ca/P molar ratio (1.67), indicating that the tin deposition did not involve an exclusive substitution of Ca^2+^ ions and a part of Sn^2+^ was likely complexed at the hydroxyapatite surface. In addition, HAADF-STEM/EDX mapping (Fig. S.1) also confirmed that there was a Sn homogeneous dispersion on the HAP surface.

Most relevant surface properties were measured. Specific surface area and pore volume values (Table S.4) are in agreement with those already reported for Sn/HAP materials in previous works (ca. 65 m^2^ g^−1^ and 0.2 cm^3^ g^−1^)^30, 31^. Point of zero charge (PZC) value, evaluated by salt addition method, results equal to ca. 6.

### Hexavalent chromium removal by Sn/HAP

In the first instance and to discriminate the effect of each metal ion, reductive adsorption tests were carried out in eight binary solutions containing potassium bichromate (Cr(VI), 20 mg L^−1^) and one of the metal salts at a time in concentration from 20 to 200 mg L^−1^.

**Figure 1:**
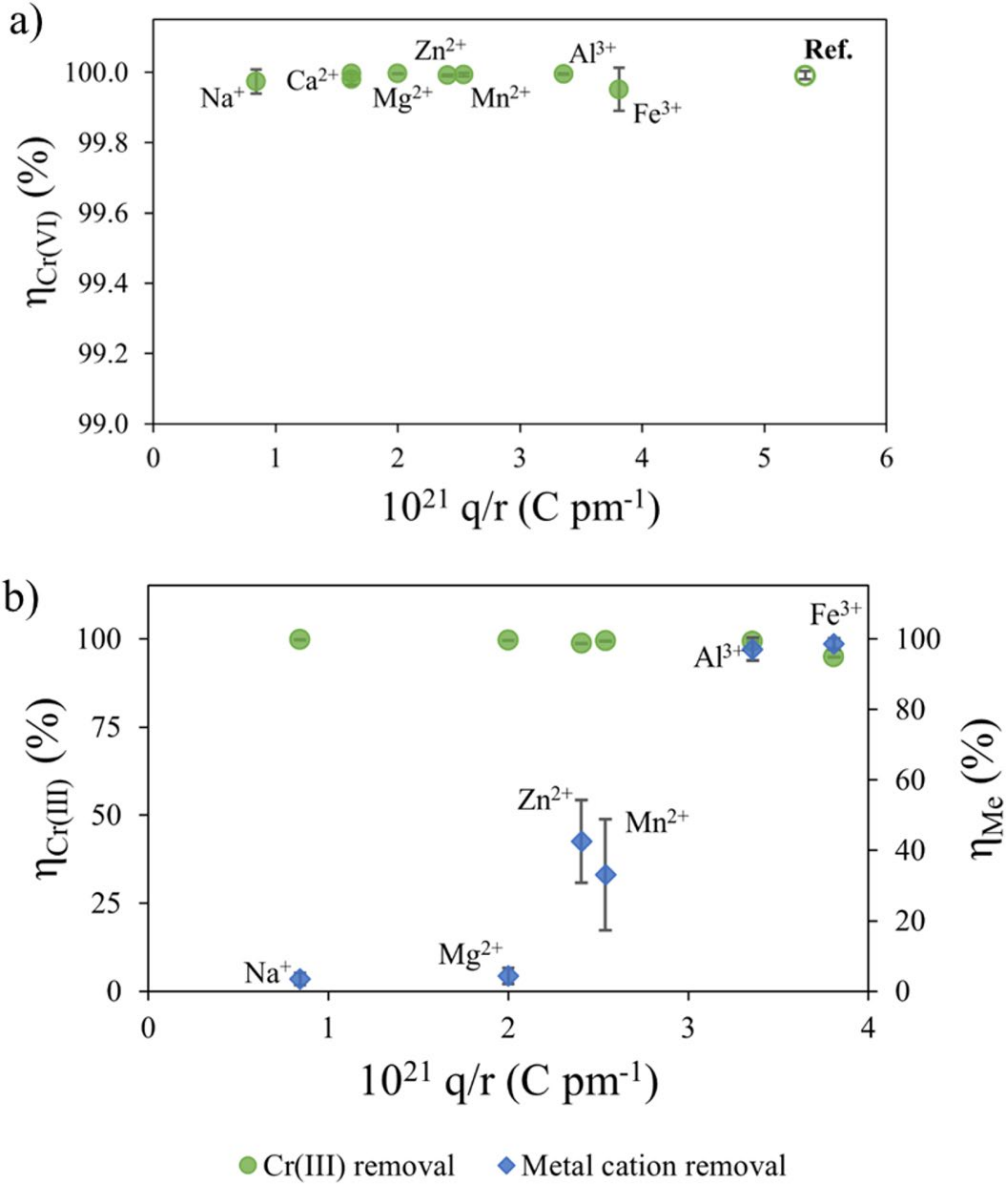
Removal efficiencies (*η*) as a function of the charge-to-radius ratio, *q/r* (where *r* is the atomic radius expressed in pm) for: (**a**) Cr(VI) and (**b**) Cr^3+^ (green markers) and the other metal ions (blue markers). The empty circle (Ref.) represents the amount of Cr(VI) removed from aqueous solution without any added salts. Bars represent standard deviations of duplicated tests. Experimental conditions: [Cr(VI)]° ~ 20 mg L^−1^; the initial concentrations of the other metal ions are reported in Table S.2; pH = 2.0; *T* = 25.0 °C; dosage = 4.5 g L^−1^; *t* = 2 h.

The computed values of removal efficiency of Cr(VI) (*η*) are reported as a function of the charge-to-radius ratio (*q/r*) of the co-present metal ions, as shown in Fig. 1a.

A first look to Fig. 1a evidences that the Cr(VI) removal efficiency of Sn/HAP was always higher than 99.8%, independently of the presence of alkaline, alkaline earth, heavy metal ions or anions. This result highlights that the co-presence of other ions did not affect the reducing capacity of Sn/HAP that is able to selectively reduce Cr(VI) even in the presence of other ion species. This is an intriguing aspect from an application point of view, as well as an unexpected result, in particular as regards the non-influence by the anions. In fact, while it has been observed in the literature that increasing the solution ionic strength has no effect on the reduction of Cr(VI) on reductive adsorbents, with reducing species different from Sn^2+^, co-existing anions could adsorb forming complexes at the interface, competing with Cr(VI) for interaction with reducing sites and also inhibiting the electron transfer.

Although the reduction of Cr(VI) was not influenced by the presence of other metal ions, the latter might interfere on the simultaneous adsorption of formed Cr^3+^, which constitutes the second step of reductive adsorption. Moreover, some ICP-MS analyses on digested Sn/HAP samples after use confirmed the total uptake of the formed Cr^3+^. Then, *η* values for Cr^3+^ close to 100% were obtained, in any case, in agreement to our previous results where solutions containing only Cr(VI) were studied. This confirms the capability of HAP to adsorb trivalent metal cations^43^.

Besides the adsorption of Cr^3+^ formed from Cr(VI) reduction, Sn/HAP, in particular the surface of HAP, might be able to adsorb the other metal cations present in the binary solutions. Therefore, removal efficiencies (*η*) of Sn/HAP towards the other metal ions present in solution with Cr(VI) were also determined (Eq. 1). The results are shown in Fig. 1b as a function of the charge-to-radius ratio (*q/r*) of the metal ions.

Sn/HAP proved to be able to adsorb almost quantitatively Fe^3+^ and Al^3+^ trivalent cations leading to high values of *η* close to 100% (Fig. 1b). Conversely, the removal efficiencies of alkaline and alkaline earth metal ions, such as Na^+^ and Mg^2+^, were extremely low (less than 5%, Fig. 1b). This observation compares well with the lack of adsorption of alkaline metal cations on hydroxyapatite reported in the literature^44^. An intermediate behaviour was observed for the divalent cations, Zn^2+^ and Mn^2+^, for which efficiencies were around 50%. According to Oliva et al.^45^, the good affinity of hydroxyapatite towards Zn^2+^ and Mn^2+^could be due to the possibility for the adsorbed metal ion to grow as stable crystalline metal phosphates, hopeite, Zn_3_(PO_4_)_2_·4H_2_O, and metaswitzerite Mn_3_(PO_4_)_2_·4H_2_O, respectively.

These first results show the good performances of Sn/HAP in the reductive adsorption of Cr(VI) and in adsorption of some other cations. This could have practical consequences for the remediation of Cr(VI) contamination from polluted waters containing different metal ions.

### Kinetics in multicomponent solution

The reductive adsorption kinetics of Cr(VI) was investigated in a multi-metallic solution containing all the studied metal ions. The kinetic test was performed at 25 °C in batch condition starting from a solution containing 20 mg L^−1^ of Cr(VI) and all the other metal ions at the same concentration as the experiments with binary solutions.

**Figure 2:**
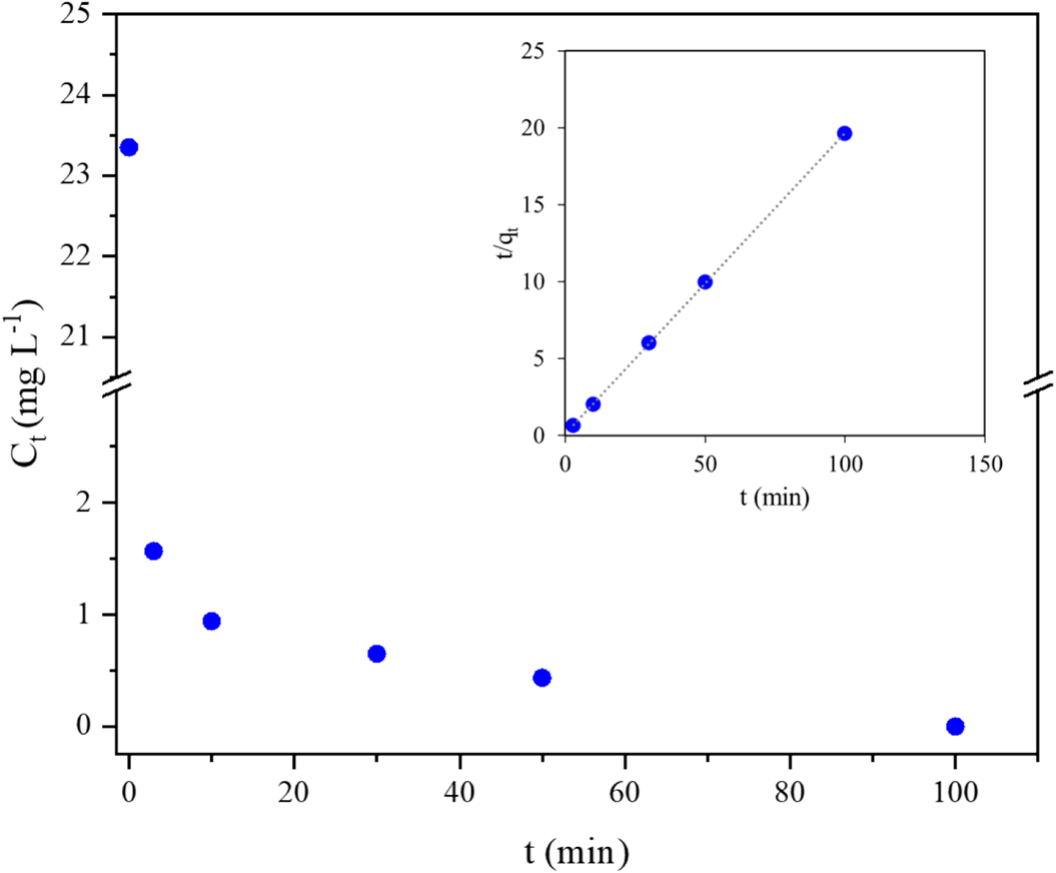
Kinetics of Cr(VI) reductive adsorption by Sn/HAP at 25.0 °C in co-presence of all studied cations; inset: fitting of experimental data with the integrated linearized form of pseudo-second order (PSO) model (see Eq. 5).

The obtained kinetic profile is shown in Fig. 2 as the residual concentration of Cr(VI) at different time. A pronounced exponential decay in Cr(VI) concentration was observed, with a steep drop from ca. 20 to ca. 2 mg L^−1^ in the first 3 min. A very fast initial reduction activity of Sn/HAP was observed with a Cr(VI) removal mean rate of ca. 6 mg L^−1^ min^−1^.

The most conventional kinetic models^38^, i.e. the PFO, PSO, and Elovich equations (see Experimental section) were used to fit the experimental data of Fig. 2. The kinetic parameters and relative statistical metrics of each model are summarised in Table S.5 with the regression plots reported in Fig. S.2. The PSO and Elovich models were found to fit at best the experimental data, as shown by the regression parameters (R^2^ and Akaike Information Criterion)^46^.

The calculated rate constant for PSO (*k*_*2*_) was 0.38 g∙mg^−1^∙min^−1^ (corresponding to 28.6 M^−1^ s^−1^); it indicates a very fast reaction rate that proceeded under the assumed conditions of the PSO model (i.e., low initial concentration for Cr(VI) and a surface rich in active sites for the adsorbent). Remarkably, the rate constant (*k*_*2*_) is twenty times higher than in the absence of co-ions (0.017 g mg^−1^ min^−1^)^31^. Such an increase, that resulted from the simultaneous presence of metal ions, has been also reported in the literature for nZVI-Fe_3_O_4_ composites in the presence of divalent cations (in this case, *k*_*2*_ increases from 0.044 to 0.528 g mg^−1^ min^−1^)^47^ and for Fe^2+^-containing clay minerals in admixture with Fe^3+^(oxyhydr)oxides^48^. According to the cited references, such an enhancement in the reductive adsorption kinetics could be due to the partial mitigation of negative surface charge of Sn/HAP by the adsorbed bivalent and trivalent cations, favoring the interaction with negatively charged Cr(VI) anions with the surface.

### Reuse of Sn/HAP in Cr(VI) reductive-adsorption tests

After the first use of the Sn/HAP sample in the reductive adsorption of 20 mg L^−1^ of Cr(VI) it is worthwhile to explore the prospect of further extending the use of Sn/HAP in several successive runs to reduce Cr(VI) and adsorb Cr^3+^ and other metal ions present in the solution. Reuse tests of Cr(VI) reductive adsorption were then performed up to saturation of the sample surface, both in terms of reductive Sn^2+^ sites (until their complete conversion to inactive Sn^4+^ sites) and adsorption capacity toward the various metal ions in solution.

**Figure 3:**
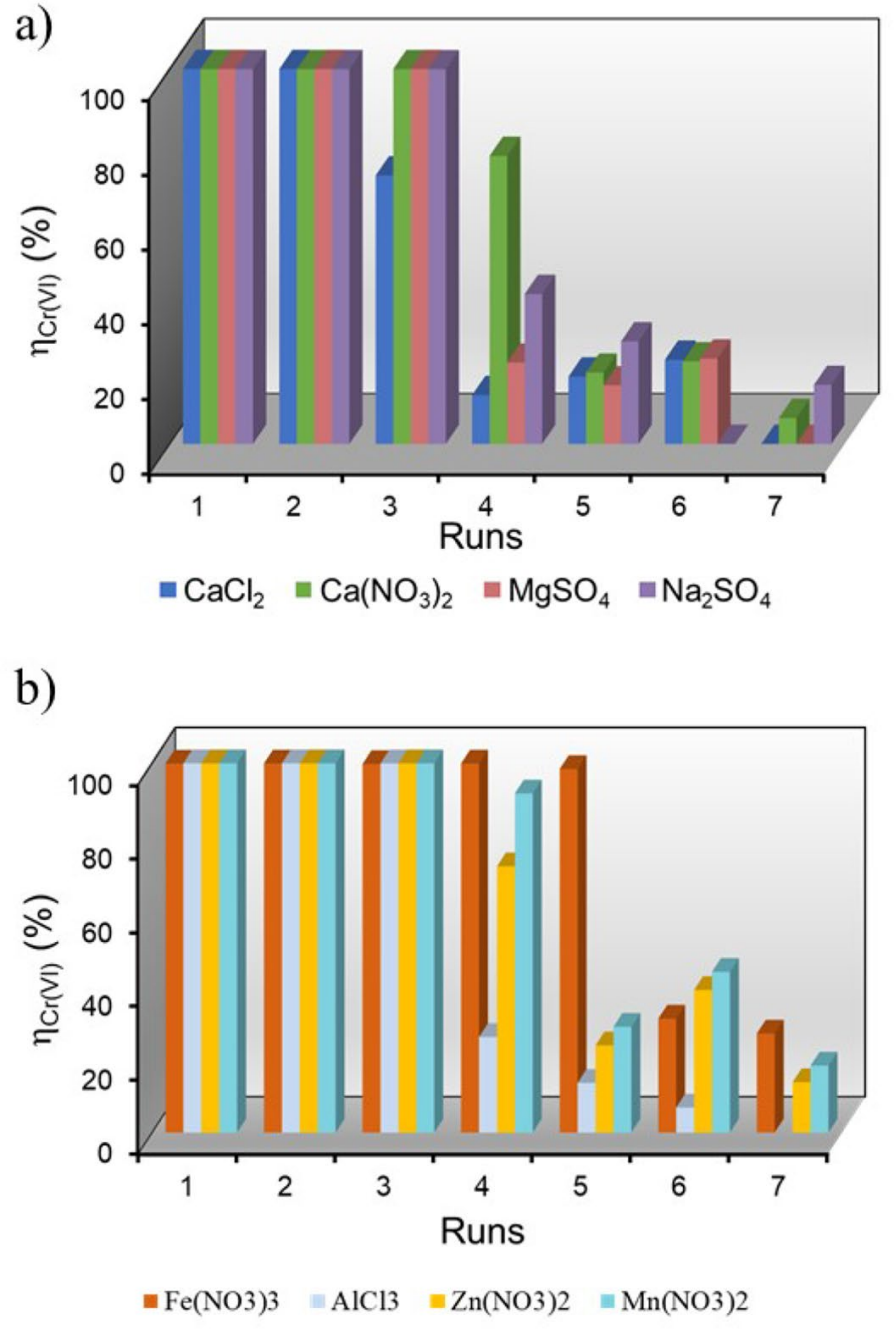
Cr(VI) removal efficiencies by Sn/HAP over successive runs in co-presence of a) alkaline and alkaline earth metal cations and b) transition metal ions. Experimental conditions: [Cr(VI)]^0^ ~ 20 mg L^−1^, the initial concentrations of the other metal ions are reported in Table S.2; pH = 2.0; *T* = 25.0 °C; *t* = 2 h. All the reductive adsorption experiments were performed in duplicate.

Reuse of the Sn/HAP sample in further runs of Cr(VI) reductive adsorption was accomplished by recovering and reusing the sample after its first use under the same experimental conditions used in the previous test. The observed Cr(VI) removal efficiency (*η*), evaluated in the presence of other metal cations (binary solutions as described in Table S.2), is shown in Fig. 3a (alkali and alkaline earth metal ions) and in (b) (transition metal ions) as a function of the number of runs.

The effect of co-existing cations on Cr(VI) removal efficiency is negligible within the first three consecutive runs (Fig. 3a,b). Regardless of the salt added to the Cr(VI) solution, Sn/HAP was able to remove nearly 100% of the Cr(VI) in the first three runs (Table 1), except for a slight decrease to 71.6% in the third run observed in the simultaneous presence of CaCl_2_. The presence of chlorides (about 360 mg L^−1^) could affect the surface charge distribution of Sn/HAP and the speciation of Cr^3+^ in solution. As reported in the literature^47^, the repulsive forces of large anions adsorbed at the interface could repel the approaching Cr(VI) ions, limiting their interaction with the reduction centres.

**Table 1:** Results of reductive adsorption tests by Sn/HAP: successive runs in co-presence of alkaline, earth alkaline and transition metal salts.

Metal salts	Ionic strength^**a**^ (mol L^−1^)		Cumulative removal capacity of Cr(VI) and other metal ions in solution (mmol g^−1^) Run						
			1	2	3	4	5	6	7
CaCl_2_^**b**^	0.018	Cr(VI)	0.0949 (100%)^**c**^	0.190 (100%)	0.258 (72%)	0.270 (13%)	0.287 (18%)	0.308 (22%)	0.308 (< 1%)
Ca(NO_3_)_2_^**b**^	0.021	Cr(VI)	0.0690 (100%)	0.138 (100%)	0.207 (100%)	0.260 (77%)	0.273 (19%)	0.288 (22%)	0.293 (7%)
MgSO_4_	0.023	Cr(VI)	0.0743 (100%)	0.149 (100%)	0.223 (100%)	0.239 (22%)	0.251 (16%)	0.268 (23%)	0.265 (< 1%)
		Mg^2+^	0.0805 (6%)	0.152 (5%)	0.250 (7%)	0.313 (5%)	0.357 (3%)	0.338 (< 1%)	n.d
Na_2_SO_4_	0.018	Cr(VI)	0.0755 (100%)	0.151 (100%)	0.227 (100%)	0.257 (40%)	0.278 (27%)	n.d	0.289 (16%)
		Na^+^	0.0425 (2%)	0.104 (3%)	0.137 (2%)	0.199 (3%)	0.232 (2%)	n.d	n.d
Fe(NO_3_)_3_	0.021	Cr(VI)	0.0727 (100%)	0.145 (100%)	0.218 (100%)	0.291 (100%)	0.362 (98%)	0.385 (31%)	0.404 (27%)
		Fe^3+^	0.621 (100%)	1.232 (98%)	1.826 (95%)	2.372 (88%)	2.389 (3%)	2.164 (< 1%)	n.d
AlCl_3_	0.023	Cr(VI)	0.0788 (100%)	0.158 (100%)	0.237 (100%)	0.257 (26%)	0.268 (13%)	0.273 (7%)	n.d
		Al^3+^	0.579 (99%)	1.086 (87%)	1.372 (49%)	1.080 (< 1%)	n.d	n.d	n.d
Zn(NO_3_)_2_	0.010	Cr(VI)	0.0772 (100%)	0.154 (100%)	0.232 (100%)	0.287 (72%)	0.305 (24%)	0.335 (39%)	0.346 (14%)
		Zn^2+^	0.160 (51%)	0.221 (19%)	0.277 (18%)	0.285 (3%)	0.245 (< 1%)	n.d	n.d
Mn(NO_3_)_2_	0.007	Cr(VI)	0.0790 (100%	0.158 (100%)	0.237 (100%)	0.310 (92%)	0.332 (29%)	0.367 (44%)	0.381 (18%)
		Mn^2+^	0.0416 (52%)	0.0656 (30%)	0.0855 (25%)	0.106 (25%)	0.109 (4%)	0.0805 (< 1%)	n.d

Experimental conditions: pH = 2.0; *T* = 25.0 °C; *t* = 2 h; dosage = 4.5 g L^−1^; nominal initial concentrations are reported in Table S.2.

All the reductive adsorption experiments were performed in duplicate.

^**a**^Calculated by VisualMINTEQ software at pH = 2.

^**b**^Cumulative removal capacity of Ca^2+^ is not reported, see the text for further explanation.

^**c**^All values into brackets correspond to percent removal calculated by comparison with the maximum allowable amount that could be adsorbed in each run.

From the 4th run onward, Cr(VI) removal efficiency dropped with a varying degree of steepness depending on the type of cation present in the Cr(VI) solution. A unique behaviour was observed in the presence of Fe(NO_3_)_3_. In this case, Cr(VI) was still quantitatively reduced and removed from aqueous solutions in the 5th reuse test. The rationale behind this unusual behaviour will be discussed in the following.

To better understand the mechanisms behind these trends, adsorption capacities (*q,* see Eq. 2) were also calculated for Cr(VI) and each metal co-ion; all computed cumulative values for successive runs are listed in Table 1. It was not possible to calculate *q* values for the Ca^2+^ ions (in the case of solutions containing Ca(NO_3_)_2_ or CaCl_2_). The partial dissolution of Sn/HAP in acidic solution (pH = 2) led to the release of Ca^2+^ ions and, consequently, to an increase in their concentration, which made it impossible to clearly evaluate the amount of calcium adsorbed (which should be calculated by the difference between the initial and final concentration of Ca^2+^ in the solution).

The results summarised in Table 1 provide interesting insights into the interactions between the Sn/HAP surface and the metal ions present in the solution. First, the alkaline and alkaline earth metals, i.e., Na^+^ and Mg^2+^, were not adsorbed by Sn/HAP in all seven consecutive tests (Table 1). Conversely, Sn/HAP was able to remove trivalent (Fe^3+^ and Al^3+^) and divalent (Zn^2+^ and Mn^2+^) metal ions. In all cases the cumulative removal capacity (*q*) passed through a maximum value in successive runs and then decreased (Table 1). This behaviour could be attributed to the inevitable partial dissolution of Sn/HAP after numerous runs in acidic solutions.

The maximum cumulative removal capacity for trivalent Fe^3+^ and Al^3+^ ions (133 mg g^−1^ and 37 mg g^−1^, corresponding to 2.39 and 1.37 mmol g^−1^, respectively) was higher than for divalent Zn^2+^ and Mn^2+^ ions (18 mg g^−1^ and 6 mg g^−1^, corresponding to 0.285 and 0.109 mmol g^−1^, respectively). Thus, it could be that Fe^3+^ and Al^3+^ ions successfully compete with Cr^3+^ for adsorption on the Sn/HAP surface, leading to premature saturation of the surface. As a result, it may no longer be possible to adsorb the formed Cr^3+^ when successive runs are performed. Conversely, Zn^2+^ and Mn^2+^ showed a low tendency to adsorb on Sn/HAP, so the surface should still be able to adsorb the formed Cr^3+^. These hypotheses are confirmed below by discussing the amounts of Cr^3+^ adsorbed in the eight reuse tests.

**Figure 4:**
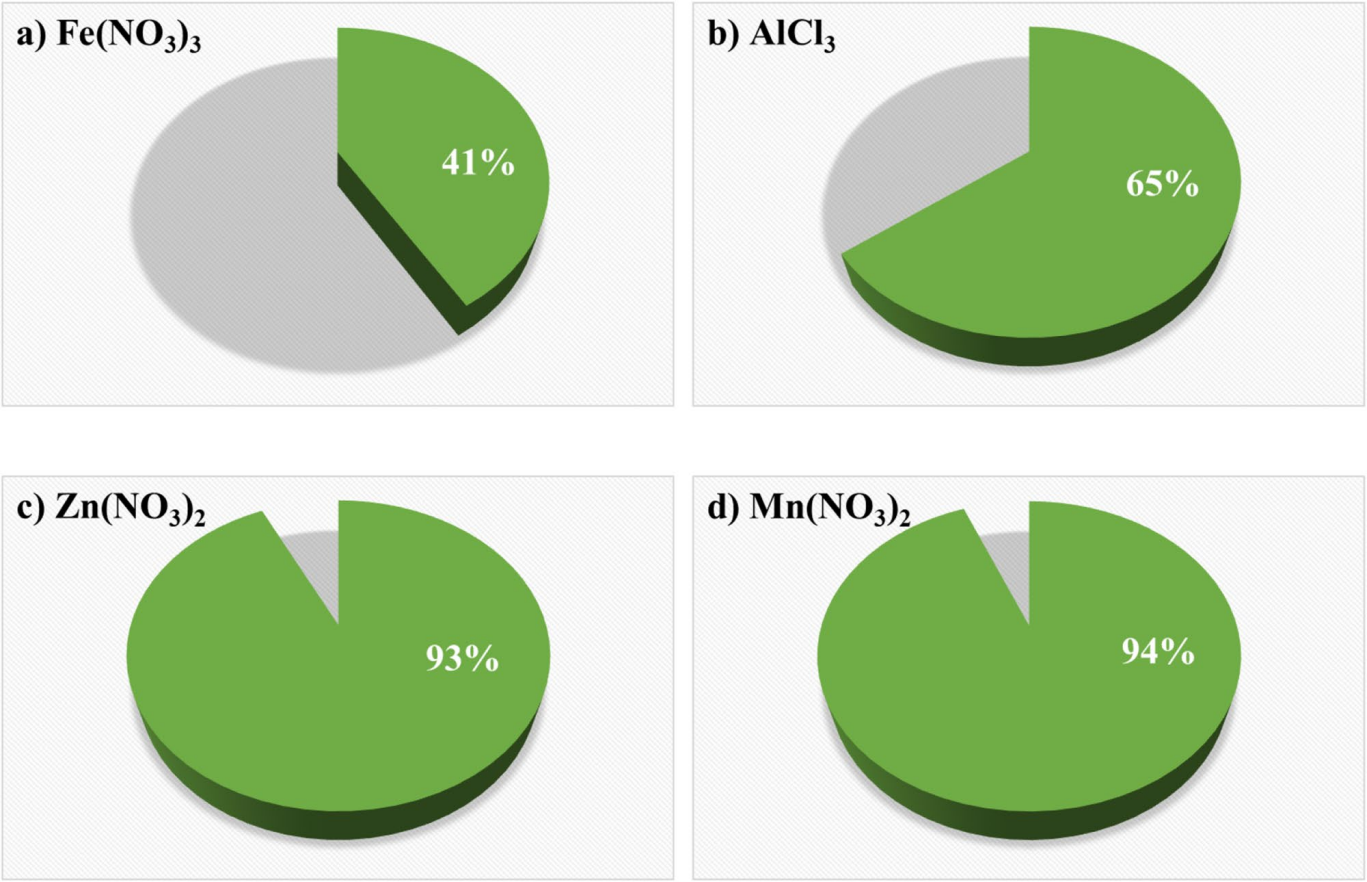
Percent of cumulative reduced Cr(VI) adsorbed on Sn/HAP as Cr^3+^ ions in the presence of (**a**) Fe(NO_3_)_3_, (**b**) AlCl_3_, (**c**) Zn(NO_3_)_2_, (**d**) Mn(NO_3_)_2_ summing up the seven successive reuse tests. Experimental conditions: [Cr(VI)]^0^ ~ 20 mg L^−1^, pH = 2.0; *T* = 25.0 °C; total time of contact of 16 h; dosage = 4.5 g L^−1^.

To demonstrate the effectiveness of the reductive adsorption process by Sn/HAP, the adsorption capacity toward the formed Cr^3+^ was measured in addition to the removal capacity of Cr(VI) (which is essentially an indicator of the reduction capacity of Sn/HAP). Table S.6 shows the comparison between removed Cr(VI) and adsorbed Cr^3+^ for the more interesting binary solutions containing Fe^3+^ or Al^3+^ or Zn^2+^ or Mn^2+^. From the data in Table S.6, the adsorbed amount of Cr^3+^ increases similarly in the first two runs in all solutions. From the 3rd run, the adsorption capacity stabilised at a value of ca. 0.2 mmol g^−1^ in the presence of Fe^3+^ or Al^3+^, while the adsorption capacity continued to increase in the presence of Zn^2+^ or Mn^2+^ until it attained a value of ca. 0.35 mmol g^−1^ after seven runs (corresponding to ca. 18 mg g^−1^). These data indicate that, as expected, the presence of trivalent cations (Fe^3+^ or Al^3+^) had a negative effect on Cr^3+^ adsorption. This information is even more evident from the aerogrammes in Fig. 4, where the total amount of Cr^3+^ adsorbed after seven reductive adsorption runs in the presence of Fe^3+^ or Al^3+^ or Zn^2+^ or Mn^2+^ is expressed as a percent with respect to the total reduced amount of Cr(VI) (i.e., the total amount of Cr^3+^ formed that could be adsorbed by Sn/HAP). As shown in Fig. 4, in the presence of divalent cations (i.e., Zn^2+^ and Mn^2+^), almost all of the reduced Cr(VI) is adsorbed as Cr^3+^ on the hydroxyapatite surface (about 93–94%). Conversely, the presence of trivalent cations (i.e., Fe^3+^ and Al^3+^) hinders the adsorption of formed Cr^3+^: only 41% and 65%, respectively, of the total reduced Cr(VI) is adsorbed on Sn/HAP as Cr^3+^. This evidence confirms the competition of trivalent cations in adsorption on Sn/HAP, especially on the hydroxyapatite surface.

### Adsorption isotherms and surface characterization

To confirm the hypothesis of competition between trivalent cations and Cr^3+^ in adsorption on Sn/HAP, adsorption isotherms of Cr^3+^ and Fe^3+^ were collected with the aim of estimating the maximum quantities of the two ions that could be adsorbed on Sn/HAP. It was preferred to study the adsorption of Fe^3+^ instead of Al^3+^ because Fe^3+^ has a stronger influence than Al^3+^ on the adsorption of Cr^3+^ (Fig. 4a).

The adsorption isotherms of Cr^3+^ and Fe^3+^ ions on Sn/HAP have been collected carrying out batch adsorption tests at pH = 2.0 and at 25.0 ± 0.5 °C under magnetic stirring condition and at a contact time of 2 h.

**Figure 5:**
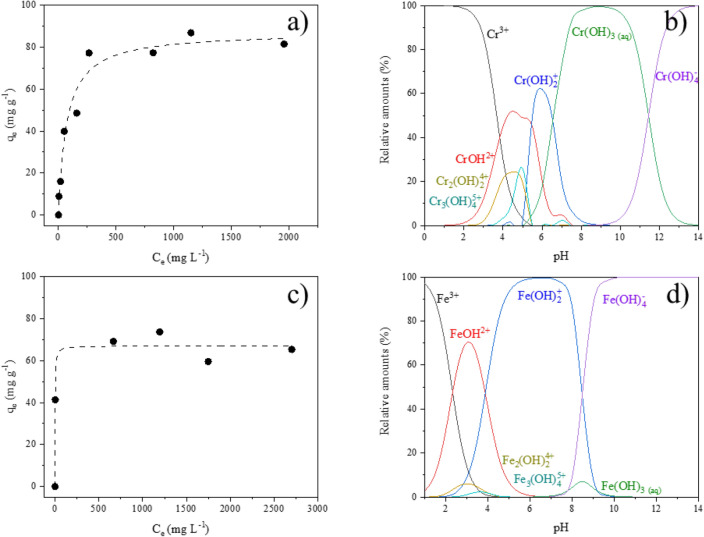
Adsorption isotherms of Cr^3+^ (**a**) and Fe^3+^ (**c**) ions onto Sn/HAP in aqueous suspensions at pH = 2.0 collected at 25.0 °C and relative distribution of Cr^3+^ (**b**) and Fe^3+^ (**d**) species as a function of pH (calculated by Visual MINTEQ software). Experimental data fitted with Langmuir isotherm equation (dashed line).

The collected experimental data (*q*_*e*_ vs. *C*_*e*_) of adsorption of Cr^3+^ and Fe^3+^ on Sn/HAP are shown in Fig. 5a–c. Both plots describe concave curves with typical Langmuir’s profile characterized by an inflection (“knee”) followed by a strict asymptotic plateau, which indicates a progressive saturation of the surface.

The Langmuir model was then used to fit the experimental data; the fitting parameters of the isotherms were calculated and reported in Table 2. The fitting curve for Cr^3+^ adsorption slightly differs from that describing Fe^3+^ adsorption (dotted lines in Fig. 5a–c panels). The initial part of the Fe^3+^ adsorption isotherm has a very high slope, indicating that iron ions have such high affinity towards Sn/HAP surface that at low concentration they are completely adsorbed, or at least no measurable amount could be detected. The isotherm curve in the case of Cr^3+^ has a less pronounced “knee” and saturation is reached above 250 mg L^−1^.

**Table 2:** Langmuir isotherm parameters for the adsorption of Cr^3+^ and Fe^3+^ ions on Sn/HAP at pH = 2 and at *T* = 25 °C.

Metal ion	q_max_ (mg g^−1^)	K_L_ (L mg^−1^)	R^2^
Cr^3+^	87.26 ± 4.33	0.014 ± 0.003	0.966
Fe^3+^	67.08 ± 2.63	0.54 ± 0.19	0.964

The maximum adsorption capacity (*q*_*max*_) of Cr^3+^ on Sn/HAP was determined to be 87.26 mg g^−1^ (corresponding to 1.68 mmol·g^−1^). This represents the maximum amount of Cr^3+^ that can be adsorbed on the Sn/HAP surface, i.e., the saturation of the Sn/HAP surface by Cr^3+^. Assuming that the total amount of Cr(VI) used in the seven reuse tests was reduced, the total adsorbable amount of Cr^3+^ formed would reach 0.54 mmol·g^−1^. This value is far below the maximum adsorption capacity on Sn/HAP, which should be able to adsorb the total amount of Cr^3+^ formed. In the presence of non-competing ions in the solution (such as Na^+^, Mg^2+^, Zn^2+^ and Mn^2+^ ions), all the Cr^3+^ formed was actually adsorbed. For example, cumulative adsorption capacity values were 0.32 and 0.36 mmol·g^−1^ for Zn^2+^ and Mn^2+^ (Table S.6), which are lower than 0.54 mmol·g^−1^ (the total adsorbable amount). This because starting from 4th run, Cr(VI) could not be completely reduced. Conversely, Cr^3+^ formed in the presence of trivalent metal ions (Fe^3+^ or Al^3+^) was not completely adsorbed.

**Figure 6:**
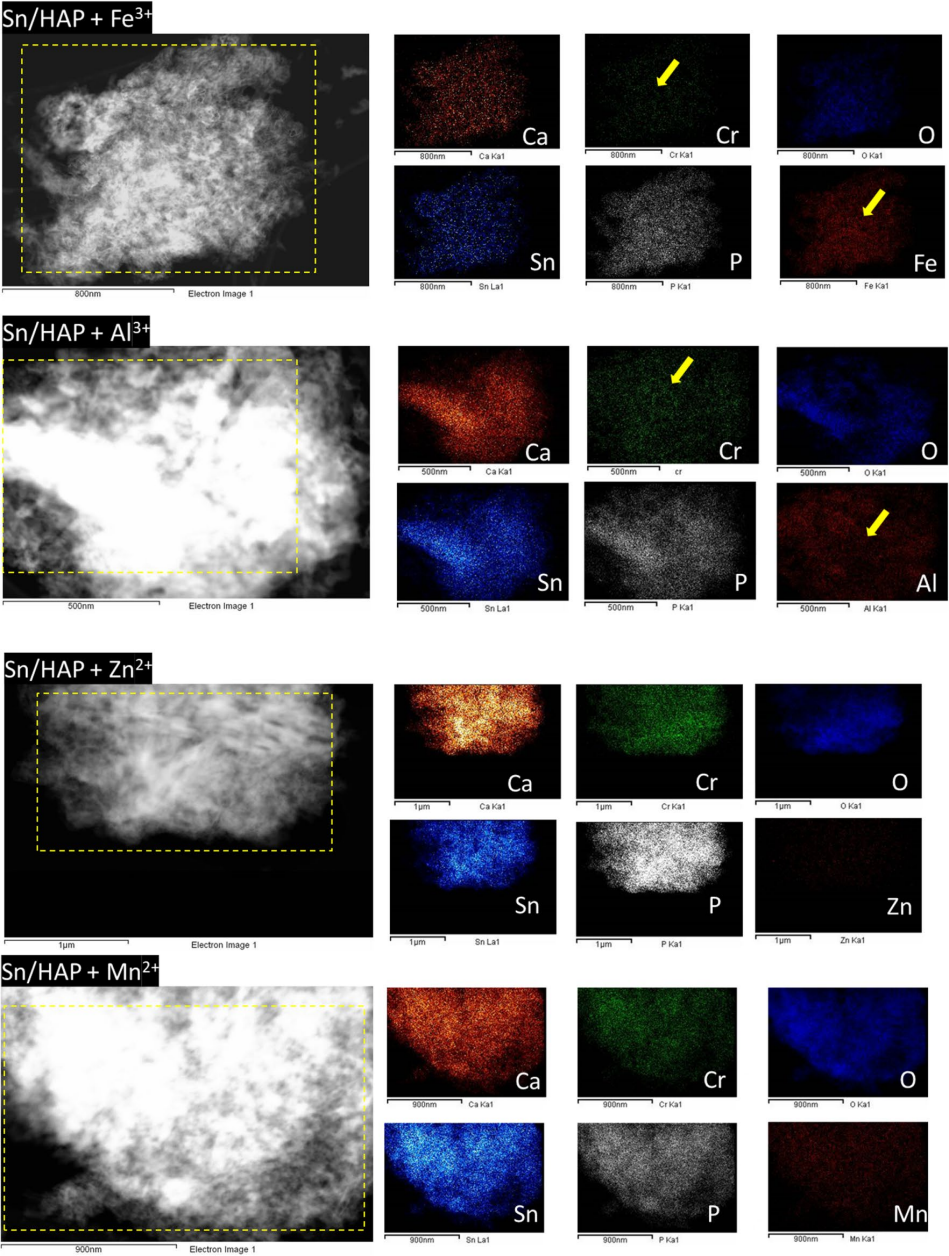
EDX compositional mapping analysis of Sn/HAP + Fe^3+^, Sn/HAP + Al^3+^, Sn/HAP + Zn^2+^ and Sn/HAP + Mn^2+^. Yellow arrows indicate a HAP zone where the evident presence of Fe^3+^ or Al^3+^ ions inhibits the deposition of Cr^3+^.

The shape of the adsorption isotherms of Fig. 5 clearly indicates a higher affinity of Fe^3+^ than Cr^3+^ for the Sn/HAP surface, and a winning competition of Fe^3+^ over Cr^3+^ for adsorption occurred. This was confirmed by computed *K*_*L*_ parameters for Cr^3+^ and Fe^3+^ whose values reflect the affinity of Sn/HAP towards the metal ions. The higher *K*_*L*_ value of Fe^3+^ (0.54 and 0.014 L mg^−1^ for Fe^3+^ and Cr^3+^, respectively) explains the unique behaviour that occurred in the reductive adsorption tests carried out in the presence of Fe(NO_3_)_3_: the Fe^3+^ ions are preferentially adsorbed on Sn/HAP than Cr^3+^, hindering the further adsorption of Cr^3+^.

For a correct interpretation of the collected data, it is important to have a clear knowledge of the speciation of metal ions in solution at specific conditions such as pH, temperature, and ionic strength. Figure 5b and d shows the relative abundance of species in solution as a function of pH for 200 mg L^−1^ Fe^3+^ ions and 200 mg L^−1^ Cr^3+^ ions as calculated by the Visual MINTEQ model analysis. The complexity of speciation for both metal ions is evident from the plots. It is noteworthy that Cr^3+^ is mainly present as an aquo-ion at pH 2, while Fe^3+^ ions and FeOH^2+^ are the predominant species at the same pH. The speciation of metal ions was also quite different in the pH range of 6–8, which is typically the interfacial pH of Sn/HAP material in contact with water. Such a relevant difference in metal speciation could help to explain the different affinity of the surface of Sn/HAP for Fe^3+^ and Cr^3+^ species.

The characterization of Sn/HAP surface after seven reuses was carried out by HAADF-STEM microscopy coupled with EDX analysis for the more relevant systems to investigate the effect of co-ions on the mechanism of the reductive adsorption process. As previously reported^31^, Cr^3+^ was well dispersed and stably anchored on Sn/HAP. Differences on the surface composition of Sn/HAP samples in the presence of trivalent (Fe^3+^ and Al^3+^) and divalent (Zn^2+^ and Mn^2+^) ions are reported and shown in Table S.7. The presence of trivalent cations limited the amount of chromium adsorbed onto Sn/HAP surface: the molar percent of superficial Cr^3+^ is smaller than that of Fe^3+^ and Al^3+^ ones (0.86 and 1.58% for Cr^3+^, 12.67 and 4.08% for Fe^3+^ and Al^3+^, respectively). These results further confirm the competition of trivalent cations with Cr^3+^, which is further evident from EDX compositional mapping analysis (Fig. 6). Conversely, divalent cations, like Zn^2+^ and Mn^2+^, did not compete with Cr^3+^ as indicated by the molar composition shown in Table S.7. In these cases, the amount of Cr^3+^ at the surface is higher than that of Zn^2+^ and Mn^2+^ ions (3.71 and 4.33% for Cr, 0.75 and 0.28% for Zn and Mn, respectively), as indicated in Fig. 6. An intriguing question concerns the knowledge about the location of the metal species adsorbed onto Sn/HAP surface. From the literature^49^, it is known that HAP is able to retain heavy metal ions through different mechanisms, e.g. surface complexation, ion exchange and dissolution–precipitation. A comparison between the STEM micrographs of fresh Sn/HAP (Fig. S.1) and used Sn/HAP (Fig. 6) shows that no difference in morphology and structuring was observed, indicating that the dissolution–precipitation mechanism could be ruled out for the adsorption of Fe^3+^, Al^3+^, Zn^2+^, and Mn^2+^. This evidence was in agreement with previous results from XRD analyses, which did not evidence the emergence of other crystalline patterns ascribable to metal oxide/phosphate phases. Otherwise, it is not possible to distinguish between the surface complexation and ion exchange mechanisms for adsorption of these metal ions on Sn/HAP. The metal cations could be allocated on different lattice positions of HAP, e.g., interstitial sites and/or Ca(1) and Ca(2) exchange sites; this is to date a critical issue still debated in the literature. In particular when Fe^3+^ adsorption on HAP materials is concerned, additional formation of small iron oxide/hydroxide clusters (with size around 2–4 nm) has been demonstrated by Mossbauer spectroscopy measurements^50^. According to the literature^51^, the presence of these clusters might be responsible for Cr(VI) adsorption, thus explaining the continuous Cr(VI) removal observed until the 5th run only in the presence of Fe^3+^ (Fig. 3).

## Conclusions

In this work, it was demonstrated that Sn/HAP can be used with success for the Cr(VI) removal in aqueous solutions in the presence of high concentration of various anions and cations which usually are co-present with hexavalent chromium in polluted waters.

Concerning anions, whatever their nature (sulphate, nitrate or chloride), they did not affect the Cr(VI) reductive adsorption. On the opposite, some cations, in particular trivalent cations (Al^3+^ and Fe^3+^), competed with Cr^3+^ for adsorption onto Sn/HAP causing a premature saturation of the surface limiting the full adsorption of Cr^3+^. The evaluation of adsorption isotherms and STEM-EDX micrographs showed that Fe^3+^ ions have a very high affinity for Sn/HAP.

While the Cr(VI) removal capacity of Sn/HAP is lower than that of other materials reported in the literature, its exceptional sustainability and straightforward preparation method position it as a promising candidate for practical applications.

A very high kinetic rate and possibility to reuse the material for several runs characterized the performances of Sn/HAP. These promising results encourage further investigation of the performance of Sn/HAP in continuous flow treatment systems using real groundwater.

### Supplementary Information

Supplementary Information.

## Data Availability

All data generated or analyzed during this study are included in this manuscript and in its supplementary information file.
