# Supplementary material for: Addressing the issue of surface mechanisms and competitive effects in Cr(VI) reductive-adsorption on tin-hydroxyapatite in the presence of co-ions
Source: Sci Rep. 2023 Nov 2;13:18913. doi: 10.1038/s41598-023-44852-7 (PMC10622583; doi:10.1038/s41598-023-44852-7)
Supplement: Supplementary file 1 — Supplementary Information. [file 41598_2023_44852_MOESM1_ESM.docx]

**Supplementary information**

**Addressing the issue of surface mechanisms and competitive effects in Cr(VI) reductive-adsorption on tin- hydroxyapatite in the presence of co-ions**

Tiziana Avola,^1^ Sebastiano Campisi,^1,^* Laura Polito,^2^ Silvia Arici,^3^ Ludovica Ferruti,^3^ Antonella Gervasini,^1,^*

**Table S.1.** Typical composition of a groundwater kindly furnished by A2A Company.

| ELEMENT | Average composition (%) |
| --- | --- |
| Calcium | 70.62 |
| Magnesium | 18.35 |
| Sodium | 9.81 |
| Iron | 0.84 |
| Aluminum | 0.12 |
| Hexavalent chromium | 0.03 |
| Total chromium | 0.03 |
| Manganese | 0.02 |
| Zinc | 0.18 |

**Table S.2.** Initial concentration of each anion/cation ~~i~~n the tests of Cr(VI) reductive adsorption containing 20 mg L^-1^ of Cr(VI) as initial concentration

| TEST N° | METAL SOURCE | CATION (mg L^-1^) | ANION (mg L^-1^) |
| --- | --- | --- | --- |
| 1 | CaCl_2_ | Ca^2+^  200 | Cl^-^  360 |
| 2 | Ca(NO_3_)_2_ | Ca^2+^  200 | NO_3_^-^  620 |
| 3 | MgSO_4_ | Mg^2+^  200 | SO_4_^2-^  790 |
| 4 | Na_2_SO_4_ | Na^+^  200 | SO_4_^2-^  420 |
| 5 | Fe(NO_3_)_3_ | Fe^3+^  200 | NO_3_^-^  670 |
| 6 | AlCl_3_ | Al^3+^  100 | Cl^-^  400 |
| 7 | Zn(NO_3_)_2_ | Zn^2+^  100 | NO_3_^-^  190 |
| 8 | Mn(NO_3_)_2_ | Mn^2+^  20 | NO_3_^-^  45 |

**Table S.3.** Measured initial and final pH values in the Cr(VI) reductive adsorption at 25.0°C in co-presence of other metal ions.

|  | **N° test** | **1** | **2** | **3** | **4** | **5** | **6** | **7** |
| --- | --- | --- | --- | --- | --- | --- | --- | --- |
| Cr(VI) CaCl_2_ | pH_i_ | 2.3 | 2.3 | 2.3 | 2.3 | 2.3 | 2.3 | 2.3 |
|  | pH_f_ | 4.7 | 4.4 | 4.3 | 4.2 | 3.8 | 2.2 | 2.1 |
| Cr(VI) Ca(NO_3_)_2_ | pH_i_ | 2.0 | 2.0 | 2.0 | 2.0 | 2.0 | 2.0 | 2.0 |
|  | pH_f_ | 4.7 | 4.4 | 4.2 | 4.1 | 3.8 | 2.2 | 2.2 |
| Cr(VI) MgSO_4_ | pH_i_ | 2.1 | 2.1 | 2.1 | 2.1 | 2.1 | 2.1 | 2.1 |
|  | pH_f_ | 4.3 | 4.5 | 4.4 | 4.4 | 3.9 | 2.2 | 2.1 |
| Cr(VI) Na_2_SO_4_ | pH_i_ | 2.1 | 2.1 | 2.1 | 2.1 | 2.1 | - | 2.1 |
|  | pH_f_ | 4.6 | 4.6 | 4.5 | 4.3 | 3.9 | - | 2.2 |
| Cr(VI) Fe(NO_3_)_3_ | pH_i_ | 2.1 | 2.1 | 2.1 | 2.1 | 2.1 | 2.1 | 2.1 |
|  | pH_f_ | 3.0 | 2.9 | 2.2 | 2.0 | 1.9 | 1.8 | 1.9 |
| Cr(VI) AlCl_3_ | pH_i_ | 2.1 | 2.1 | 2.1 | 2.1 | 2.1 | 2.1 | 2.1 |
|  | pH_f_ | 4.4 | 3.0 | 2.7 | 2.3 | 2.1 | 2.0 | 2.1 |
| Cr(VI) Zn(NO_3_)_2_ | pH_i_ | 2.2 | 2.2 | 2.2 | 2.2 | 2.2 | 2.2 | 2.2 |
|  | pH_f_ | 4.2 | 3.9 | 3.8 | 3.6 | 3.4 | 2.2 | 2.0 |
| Cr(VI) Mn(NO_3_)_2_ | pH_i_ | 2.1 | 2.1 | 2.1 | 2.1 | 2.1 | 2.1 | 2.1 |
|  | pH_f_ | 4.5 | 4.3 | 4.2 | 4.0 | 3.6 | 2.2 | 2.0 |

**Table S.4.** Composition and properties of Sn/HAP batches used in the reductive adsorption tests.

| Sample code**^a^** | Sn content**^b^**  (wt. %) | (Ca + Sn)/P ratio**^b^** | Surface area**^c^**  (m^2^ g^-1^) | Pore volume**^d^**  (cm^3^ g^-1^) |
| --- | --- | --- | --- | --- |
| Sn/HAP | 13.39 ± 0.79 | 2.27 ± 0.18 | 64.5± 0.8 | 0.24 ± 0.01 |
| **^a^** Surface area of 78 m^2^ g^-1^ and pore volume of 0.25 cm^3^ g^-1^ for bare HAP ^31^  **^b^** Average value determined by ICP-MS of different prepared batches of Sn/HAP samples.  **^c^** Evaluated according to 3-parameters BET model.  **^d^** Evaluated according to B.J.H. model. | | | | |

**Table S.5.** Computed kinetic parameters of the Cr(VI) reductive adsorption at 25.0°C in co-presence of other metal ions by using the PFO, PSO and Elovich models.

| PFO model | | |  | PSO model | | |  | Elovich model | | |  |
| --- | --- | --- | --- | --- | --- | --- | --- | --- | --- | --- | --- |
| k_1_**^a^** | q_e_**^b^** | R^2^ | AIC_C_^c^ | k_2_**^d^** | q_e_**^b^** | R^2^ | AIC_C_^c^ | a**^e^** | b**^f^** | R^2^ | AIC_C_^c^ |
| 0.031±0.005 | 0.32±0.05 | 0.946 | 25.6 | 0.38±0.17 | 5.11±0.02 | 0.999 | -7.2 | 51.32 | 10.54 | 0.985 | -32.3 |
| **^a^** min^-1^;  **^b^** mg g^-1^;  **^c^** g mg^-1^ min^-1^;  ^d^ Akaike Information Criterion: AIC = $2K+N\ln\left[ \frac{\mathrm{SSE}}{N} \right]+\frac{2K(K+1)}{N-K-1}$, where K is the number of parameters of the model, N is the number of experimental points, and SSE is the sum of squared errors SSE = $\sum_{i}^{N} \left( q_{t}^{\exp}-q_{t}^{\mathrm{mod}} \right)_{i}^{2}$  **^e^** mg g^-1^ min^-1^;  **^f^** g mg^-1^ | | | | | | | | | | |  |

**Table S.6.** Comparison of Cr(VI) and Cr^3+^ removed by Sn/HAP in co-presence of transition metal cations.

| Metal ions | Fe(NO_3_)_3_ | | AlCl_3_ | | Zn(NO_3_)_2_ | | Mn(NO_3_)_2_ | |
| --- | --- | --- | --- | --- | --- | --- | --- | --- |
| N° test | Cumulative removal capacity (*q_e_*)**^a^** | | | | | | | |
|  | Cr(VI) | Cr^3+^ | Cr(VI) | Cr^3+^ | Cr(VI) | Cr^3+^ | Cr(VI) | Cr^3+^ |
| 1 | 0.0727 | 0.0690 | 0.0788 | 0.0782 | 0.0772 | 0.0762 | 0.0790 | 0.0785 |
| 2 | 0.145 | 0.135 | 0.158 | 0.144 | 0.154 | 0.153 | 0.158 | 0.158 |
| 3 | 0.218 | 0.180 | 0.237 | 0.211 | 0.232 | 0.229 | 0.237 | 0.235 |
| 4 | 0.291 | 0.213 | 0.257 | 0.206 | 0.287 | 0.283 | 0.310 | 0.306 |
| 5 | 0.362 | 0.203 | 0.268 | 0.196 | 0.306 | 0.296 | 0.332 | 0.328 |
| 6 | 0.385 | 0.187 | 0.273 | 0.184 | 0.335 | 0.321 | 0.367 | 0.352 |
| 7 | 0.404 | 0.167 | - | - | 0.346 | 0.321 | 0.381 | 0.358 |
| **^a^** expressed as mmol g^-1^ | | | | | | | | |
| **Table S.7.** Composition of the used samples determined by HAADF-STEM/EDX mapping.   \|  \| Fe+Cr/SnHAP \| Al+Cr/SnHAP \| Zn+Cr/SnHAP \| Mn+Cr/SnHAP \| \| --- \| --- \| --- \| --- \| --- \| \| Element \| EDX Composition mol. (%) \| \| \| \| \| P \| 18.46 \| 25.44 \| 19.99 \| 18.72 \| \| Ca \| 1.27 \| 7.25 \| 4.19 \| 1.76 \| \| Sn \| 4.23 \| 23.06 \| 30.80 \| 18.17 \| \| O \| 62.52 \| 38.60 \| 40.56 \| 56.73 \| \| Cr \| 0.86 \| 1.58 \| 3.71 \| 4.33 \| \| Fe \| 12.67 \| - \| - \| - \| \| Al \| - \| 4.08 \| - \| - \| \| Zn \| - \| - \| 0.75 \| - \| \| Mn \| - \| - \| - \| 0.28 \|   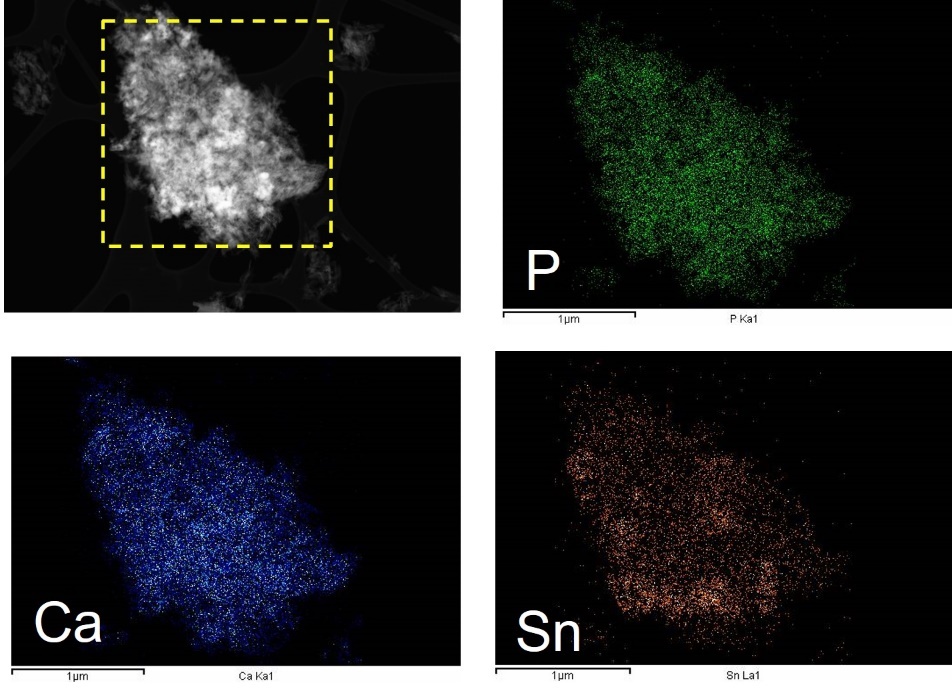  **Figure S.1.** EDX compositional mapping analysis of Sn/HAP sample. | | | | | | | | |


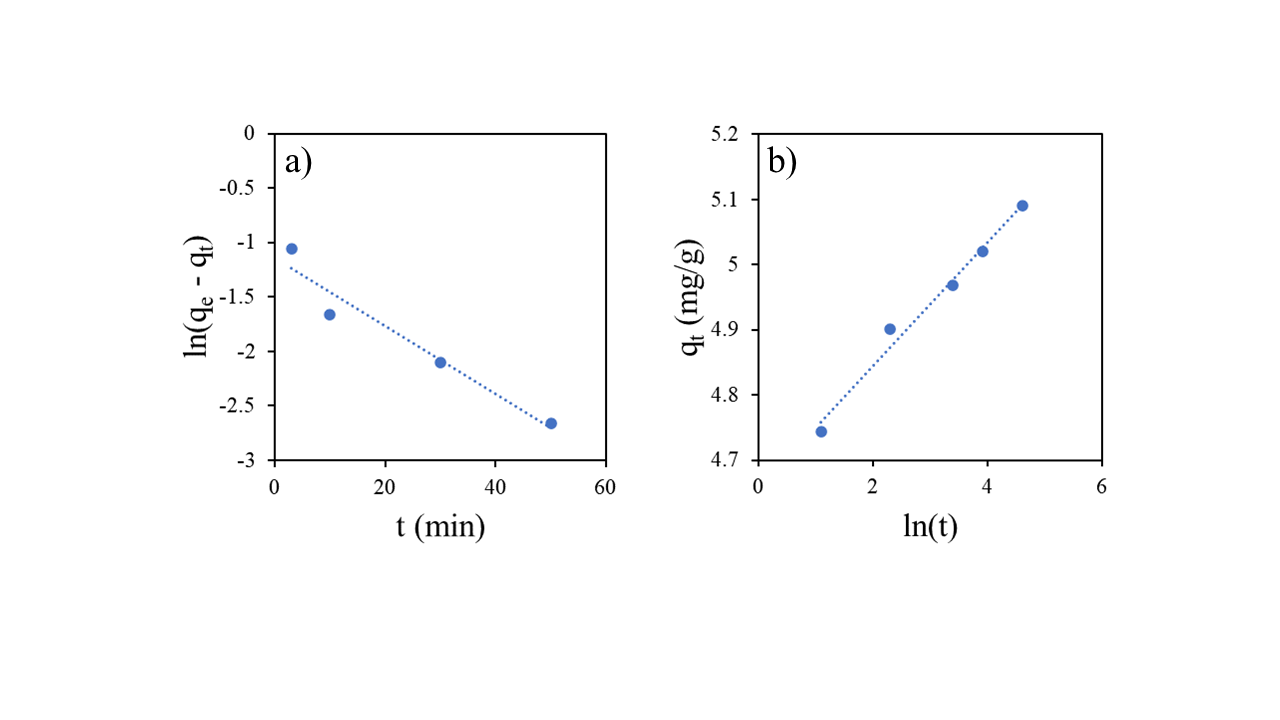


**Figure S.2.** Fitting of experimental data with the integrated linearized form of pseudo-first order (PFO) (a) and Elovich models (b).
